# Bayesian Optimization for Neuroimaging Pre-processing in Brain Age Classification and Prediction

**DOI:** 10.3389/fnagi.2018.00028

**Published:** 2018-02-12

**Authors:** Jenessa Lancaster, Romy Lorenz, Rob Leech, James H. Cole

**Affiliations:** ^1^Computational, Cognitive and Clinical Neuroimaging Laboratory, Division of Brain Sciences, Department of Medicine, Imperial College London, London, United Kingdom; ^2^Department of Neuroimaging, Institute of Psychiatry, Psychology and Neuroscience, King’s College London, London, United Kingdom

**Keywords:** brain aging, Bayesian optimization, T1-MRI, machine learning, pre-processing

## Abstract

Neuroimaging-based age prediction using machine learning is proposed as a biomarker of brain aging, relating to cognitive performance, health outcomes and progression of neurodegenerative disease. However, even leading age-prediction algorithms contain measurement error, motivating efforts to improve experimental pipelines. T1-weighted MRI is commonly used for age prediction, and the pre-processing of these scans involves normalization to a common template and resampling to a common voxel size, followed by spatial smoothing. Resampling parameters are often selected arbitrarily. Here, we sought to improve brain-age prediction accuracy by optimizing resampling parameters using Bayesian optimization. Using data on *N* = 2003 healthy individuals (aged 16–90 years) we trained support vector machines to (i) distinguish between young (<22 years) and old (>50 years) brains (classification) and (ii) predict chronological age (regression). We also evaluated generalisability of the age-regression model to an independent dataset (CamCAN, *N* = 648, aged 18–88 years). Bayesian optimization was used to identify optimal voxel size and smoothing kernel size for each task. This procedure adaptively samples the parameter space to evaluate accuracy across a range of possible parameters, using independent sub-samples to iteratively assess different parameter combinations to arrive at optimal values. When distinguishing between young and old brains a classification accuracy of 88.1% was achieved, (optimal voxel size = 11.5 mm^3^, smoothing kernel = 2.3 mm). For predicting chronological age, a mean absolute error (MAE) of 5.08 years was achieved, (optimal voxel size = 3.73 mm^3^, smoothing kernel = 3.68 mm). This was compared to performance using default values of 1.5 mm^3^ and 4mm respectively, resulting in MAE = 5.48 years, though this 7.3% improvement was not statistically significant. When assessing generalisability, best performance was achieved when applying the entire Bayesian optimization framework to the new dataset, out-performing the parameters optimized for the initial training dataset. Our study outlines the proof-of-principle that neuroimaging models for brain-age prediction can use Bayesian optimization to derive case-specific pre-processing parameters. Our results suggest that different pre-processing parameters are selected when optimization is conducted in specific contexts. This potentially motivates use of optimization techniques at many different points during the experimental process, which may improve statistical sensitivity and reduce opportunities for experimenter-led bias.

## Introduction

The aging process affects the structure and function of the human brain in a characteristic manner that can be measured using neuroimaging. This quantifiable relationship was key to the early demonstrations of voxel-based morphometry (VBM) (Good et al., 2001) and to this day represents one of the most robust relationships between a measurable phenomenon (i.e., aging) and brain structure. This makes aging an ideal target for evaluating novel neuroimaging analysis tools. More recently, researchers have used this relationship to develop neuroimaging-based tools for predicting chronological age in healthy people using machine learning (Franke et al., 2010; Cole et al., 2017b). A ‘brain-predicted age’ determined from magnetic resonance imaging (MRI) scans represents an intuitive summary measure of the natural deterioration associated with the effects of the aging process on the brain, and has the potential to serve as biomarker of age-related health (Cole, 2017).

The extent to which brain-predicted age is greater than an individual’s chronological age has been associated with accentuated age-associated physical and cognitive decline (Cole et al., 2017c). Specifically, an ‘older’-appearing brain has been associated with decreased fluid intelligence, reduced lung function, weaker grip strength, slower walking speed and an increased likelihood of mortality in older adults (Cole et al., 2017c). Factors which could contribute to an increased brain-predicted age include genetic effects, neurological or psychiatric conditions, or poor physical health (Koutsouleris et al., 2013; Löwe et al., 2016; Steffener et al., 2016; Cole et al., 2017c,d; Pardoe et al., 2017). Potentially, individuals at increased risk of experiencing the negative consequences of brain aging, such as cognitive decline and neurodegenerative disease, could be identified by measuring brain-predicted age in clinical groups or even screening the general population.

Despite promising results to-date, models for generating brain-predicted age still continue to contain measurement error, and efforts to improve accuracy and particularly, generalisability, to data from different MRI scanners are warranted. Training on large cohorts of healthy adults is one approach to reduce error, with the lowest mean absolute error (MAE) rates between 4 and 5 years (Konukoglu et al., 2013; Irimia et al., 2014; Steffener et al., 2016; Cole et al., 2017b). Notably, individual errors range across the population from perfect prediction to discrepancies as great as 25 years. While brain-predicted age has high test–retest reliability (Cole et al., 2017b), and a proportion of this variation likely reflects underlying population variability, certainly a substantial amount of noise remains. This means that a model with an MAE = 0 is highly unlikely, however, the lower bound of prediction accuracy has not yet been reached, as indicated by the gradual improvements in performance seen in more recent methods. This means that efforts to reduce noise, improve prediction accuracy and in particular the generalisability to new data is essential if such approaches are to be applied to individuals in a clinical setting, the ultimate goal of any putative health-related biomarker.

A key issue in brain-age prediction, along with many other neuroimaging approaches, is the choice of methods for extracting features or summary measures from raw data for further analysis (Jones et al., 2005; Klein et al., 2009; Franke et al., 2010; Andronache et al., 2013; Shen and Sterr, 2013). For example, the majority of brain-age prediction pipelines have used T1-weighted MRI data and either generated voxelwise maps of brain volume (e.g., Cole et al., 2015) or summary measures of cortical thickness and subcortical volumes (e.g., Liem et al., 2017). The parameters set during image pre-processing are commonly the defaults supplied by the software developer or are based on prior studies. Nevertheless, the choice of pre-processing parameters may have a strong influence on the outcome of any subsequent data modeling, and ideally should be optimized on a case-by-case basis. This optimization is rarely conducted, as trial-and-error approaches are time-consuming and often ill-posed. Importantly, using sub-optimal pre-processing may reduce experimental precision, which increases the likelihood of false positives or false negatives as well as reducing reproducibility. In the worst-case scenario, p-hacking may occur, whereby pre-processing is manually optimized based on minimizing the resultant p-values of the subsequent hypothesis testing within the same sample. Here, we outline a principled Bayesian optimization strategy for identifying optimal values for pre-processing parameters in neuroimaging analysis, implementing sub-sampling to avoid bias. We then demonstrate proof-of-principle applied to the problem of age prediction using machine learning.

Bayesian optimization is an efficient and unbiased approach to parameter selection, which avoids both the failure to adequately search a large parameter space and the drawbacks of an exhaustive search. Instead, it utilizes a guided sampling strategy, assessing a subgroup of points from within the possible parameter space and testing these values on subsets of the total sample (Brochu et al., 2010; Snoek et al., 2012). This data division strategy ensures performance tests reflect out-of-sample prediction and always evaluate differing conditions on separate data, reducing the likelihood of overfitting. This intelligent selection of a small number of points for evaluation allows the characterization of parameter space and the solution of the optimization problem to be accomplished in fewer steps, making it a computationally efficient approach (Pelikan et al., 2002).

The current work used Bayesian optimization to attempt to optimize image pre-processing parameters for: (i) distinguishing the brains of young and old adults (classification), (ii) predicting chronological age (regression), and (iii) evaluating the generalisability of the resulting optima for the regression task to an independent dataset. The classification task was included to allow evaluation of Bayesian Optimization hyper-parameters and to show the applicability of Bayesian optimization to different contexts. We hypothesized that by using Bayesian optimization we would improve model accuracy compared to previously used ‘non-optimized’ values. The study was designed to show proof-of-principle of the applicability of Bayesian optimization to help improve neuroimaging pre-processing in a principled and unbiased fashion.

## Materials and Methods

### Neuroimaging Datasets

This study used data compiled from 14 public sources (see **Table 1**), and as per our previous research (e.g., Cole et al., 2015), referred to here as the brain-age healthy control (BAHC) dataset. Data included T1-weighted MRI from 2003 healthy individuals aged 16–90 years (male/female = 1016/987, mean age = 36.50 ± 18.52). All BAHC participants were either originally included in studies of healthy individuals, or as healthy controls from case-control studies. As such, all were screened according to local study protocols to ensure they had no history of neurological, psychiatric or major medical conditions. Images were acquired at 1.5T or 3T with standard T1-weighted MRI sequences (see **Table 1**). Ethical approval and informed consent were obtained locally for each study covering both participation and subsequent data sharing.

**Table 1:** Data sources for healthy brain age training sample.

Cohort	*N*	Age mean (*SD*)	Age range	Sex male/female	Repository details	Scanner (Field strength)	Scan	Voxel dimensions
ABIDE (Autism Brain Imaging Data Exchange)	184	25.93 (6.66)	18–48	161/23	INDI	Various (all 3T)	MPRAGE	Various
Beijing Normal University	181	21.25 (1.92)	16–28	72/109	INDI	Siemens (3T)	MPRAGE	1.33 × 1.0 × 1.0
Berlin School of Brain and Mind	49	30.99 (7.08)	20–60	24/25	INDI	Siemens Tim Trio (3T)	MPRAGE	1.0 × 1.0 × 1.0
CADDementia	12	62.33 (6.26)	55–79	9/3	http://caddementia.grand-challenge.org/	GE Signa (3T)	3D IR-FSPGR	0.9 × 0.9 × 1.0
Cleveland Clinic	31	43.55 (11.14)	24–60	11/20	INDI	Siemens Tim Trio (3T)	MPRAGE	2.0 × 1.0 × 1.2
ICBM (International Consortium for Brain Mapping)	322	24.84 (5.14)	24-60	177/142	LONI IDA	Siemens Magnetom (1.5T)	MPRAGE	1.0 × 1.0 × 1.0
IXI (Information extraction from Images)	561	48.62 (16.49)	20–86	250/311	http://biomedic.doc.ic.ac.uk/brain-development	Philips Intera (3T); Philips Gyroscan Intera (1.5T); GE Signa (1.5T)	T1-FFE; MPRAGE	0.9375 × 0.93751 × 1.2
MCIC (MIND Clinical Imaging Consortium)	93	32.49 (11.95)	18–60	64/29	COINS	Siemens Sonata/Trio (1.5/3T); GE Signa (1.5T)	MPRAGE; SPGR	0.625 × 0.625 × 1.5
MIRIAD (Minimal Interval Resonance Imaging in Alzheimer’s Disease)	23	69.66 (7.18)	58–85	12/11	http://www.ucl.ac.uk/drc/research/methods/miriad-scan-database	GE Signa (1.5T)	3D IR-FSPGR	0.9375 × 0.93751 × 1.5
NEO2012 (Adelstein et al., 2011)	39	29.59 (8.38)	20–49	18/21	INDI	Siemens Allegra (3T)	MPRAGE	1.0 × 1.0 × 1.0
Nathan Kline Institute (NKI)/Rockland	160	41.49 (18.08)	18–85	96/64	INDI	Siemens Tim Trio (3T)	MPRAGE	1.0 × 1.0 × 1.0
OASIS (Open Access Series of Imaging Studies)	288	44.06 (23.04)	18–90	106/188	http://www.oasis-brains.org/	SiemensVision (1.5T)^∗^	MPRAGE	1.0 × 1.0 × 1.25
WUSL (Power et al., 2012)	24	23.04 (1.42)	20–24	4/20	INDI	Siemens Tim Trio (3T)	MPRAGE	1.0 × 1.0 × 1.0
TRAIN-39	36	22.67 (2.56)	18–28	11/25	INDI	Siemens Allegra (3T)	MPRAGE	1.33 × 1.33 × 1.3
Training set total	2003	36.50 (18.52)	16–90	1016/987	–	–	–	–

*INDI, International Neuroimaging Data-sharing Initiative (http://fcon\_1000.projects.nitrc.org).*

*COINS, Collaborative Informatics and Neuroimaging Suite (http://coins.mrn.org).*

*LONI, Laboratory of Neuro Imaging Image & Data Archive (https://ida.loni.usc.edu).*

*ABIDE consortiums comprising data from various sites with different scanners/parameters.*

*^∗^OASIS scans were acquired four times and then averaged to increase signal-to-noise ratio.*

Additionally, the Cambridge Centre for Aging Neuroscience (CamCAN) neuroimaging cohort was used as an independent validation dataset (Shafto et al., 2014; Taylor et al., 2017). These data were obtained from the CamCAN repository^1^. The CamCAN cohort consisted of T1-weighted images (acquired at 3T, using a 3D MPRAGE sequence: repetition time = 2250 ms, echo time = 2.99 ms, inversion time = 900 ms; flip angle = 9°; field of view = 256 × 240 × 192 mm; 1 mm isotropic voxels) from 648 participants aged 18–88 years (male/female = 324/324, mean age 54.28 ± 18.56). This study used similar exclusion criteria, including only healthy individuals. Ethical approval for CamCAN was obtained locally, including the permission to subsequently make anonymised versions of the data publicly available.

**FIGURE 1:**
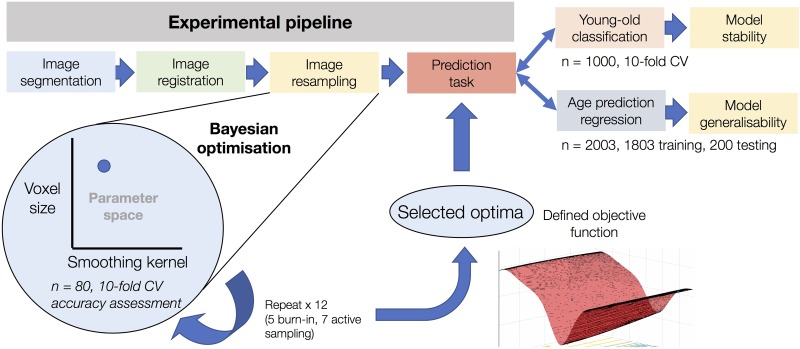
Experimental overview of the use of Bayesian optimization in image pre-processing. The figure shows the resampling stage of the experimental pipeline as the element subject to Bayesian optimization. After optimization based on 12 iterations using sub-samples on *n* = 80 and assessed using 10-fold cross-validation, the defined objective function of the corresponding parameter space can be used to determine optimal values for voxel size and smoothing kernel size. This process was conducted in a task-specific manner (i.e., once for young-old classification and once for age prediction regression), resulting in separate optima for each task. After determining model accuracy for each task, the classification model was used to determine the stability of the Bayesian optimization features and the regression model was used to determine the generalisability of the model to an entirely independent dataset.

An outline of the analysis pipeline for the study has been included as **Figure 1**. The details of each stage are as follows.

### Pre-processing to Prediction

Normalized brain volume maps were created following a standard VBM protocol, described previously (Cole et al., 2015). This involved segmentation of raw T1-weighted images into gray matter maps using SPM12 (University College London, London, United Kingdom). Images were normalized to a study-specific template in MNI152 space using DARTEL for non-linear registration (Ashburner, 2007). This step involved resampling to a common voxel size, modulation to retain volumetric information and spatial smoothing; the specific voxel size and smoothing kernel size parameters were chosen by the Bayesian optimization protocol as detailed below.

After pre-processing, gray matter volume images were converted to vectors of ASCII-format intensity values. These were used as the input features for subsequent classification or regression analysis. This was performed in MATLAB using the support vector machine (SVM) program. For the binary classification problem of distinguishing young and old participants, SVMs were used. For predicting age as a continuous variable, SVM regression (SVR) was used. Both SVM and SVR procedures used a linear kernel to map the input data into a computationally efficient feature space.

### Bayesian Optimization of Pre-processing

Bayesian optimization was used to identify optimal pre-processing parameters, based on the accuracy of the subsequent model predictions (either classification or regression). Hence, the Bayesian optimization procedure can be seen as an additional outer layer of analysis, that surrounds the standard pipeline (pre-processing through to model accuracy evaluation). The Bayesian optimization process runs multiple iterations of this internal pipeline using different sub-samples of the dataset, exploring the parameter space to select varying image pre-processing options based on their influence on the objective function (i.e., classification or regression accuracy).

A key advantage of Bayesian optimization derives from its ‘surrogate’ model that represents the relationship between an algorithm and the initially unknown objective function. This surrogate model is progressively refined in a closed-loop manner, by automatically selecting points in the parameter space. This provides an informed coverage of that space, based on the performance of previously sampled points. This aspect makes Bayesian optimization highly efficient, reducing the number of iterations necessary to identify optima of complex objective functions (Brochu et al., 2010; Lorenz et al., 2017). We used the MATLAB Bayesian Optimization Algorithm^2^ implementation, which internally defines a number of optimization parameters, including selecting the covariance kernel and tuning the hyper-parameters of the process.

In this study, we aimed to optimize the final normalized voxel size and the full-width half-maximum (FWHM) of the spatial smoothing kernel used during final resampling. Conventionally, these are set between 1 and 2 mm^3^ and either 4 or 8 mm FWHM, respectively for VBM studies conducted using SPM. In our previous work on brain-age prediction we used 1.5 mm^3^ voxel dimensions and a 4 mm smoothing kernel (Cole et al., 2015, 2017a,b,c,d; Pardoe et al., 2017), which we subsequently refer to as ‘un-optimized’ pre-processing parameters. Comparison of classification and regression accuracy was compared between optimized and un-optimized models using Steiger’s *z*-test for dependent correlations and McNemar’s chi-square for paired nominal data accordingly. Using Bayesian optimization, a wider range of values were considered; between 1 and 30 mm isotropic was permitted for voxel size and 1–20 mm FWHM for smoothing kernel size for the classification task, while ranges of 1–15 mm and 1–10 mm were used for the regression task (the latter smaller parameter space was used to reduce computation time for the more intensive task). The time taken for the optimization procedure was approximately 30 s per image for the resampling step, running on a desktop computer with 16GB RAM. A full optimization cycle thus took approximately 8 h to complete 12 iterations (see below).

### Classifying Young and Old Adults

We categorized the 500 oldest individuals (aged 51–90 years) and the 500 youngest (aged 16–22 years) as the “old” and “young” groups for classification. Each iteration of Bayesian optimization used a sub-sample of the total dataset (*N* = 1000), to test a combination of pre-processing parameter values. Participants were divided into subsets of size *n* stratified by age, such that each subset contained an approximately representative distribution of participants from across the age range, resulting in a total of *N*/*n* iterations. We used *n* = 80 total (40 participants from each group) as a sample for each iteration, giving 1000/80 = 12 iterations of Bayesian optimization. This included a burn-in phase (i.e., preliminary phase of unevaluated samples to initialize the process) of 5 randomly sampled points from within the parameter ranges to begin characterization of the search space, followed by 7 iterations of ‘guided’ active sampling. In each iteration, a voxel size and smoothing kernel size combination was selected and used for resampling during DARTEL normalization of each subject’s images. Normalized images were then converted to feature vectors and a binary SVM classifier was trained and assessed using 10-fold cross-validation. SVM hyper-parameters were left at default values. Classifier error was the objective function to be minimized. Bayesian optimization used the Expected Improvement Plus (EI+) acquisition function, with the default exploration-exploitation ratio of 0.5.

### Regression Prediction of Chronological Age

Next, we used Bayesian optimization in the context of regression models which predict chronological age in healthy individuals using brain volume images. This was done by first identifying optimal pre-processing parameters through Bayesian optimization, then applying them to the full training dataset and comparing the resulting prediction accuracy to that achieved in using ‘un-optimized’ values. The regression analysis used *n* = 80 participants per iteration, with age values spanning the full range (16–90 years) and the same Bayesian optimization hyper-parameters as above. The MAE in age prediction across 10-fold cross-validation using SVM regression (i.e., SVR) was the objective function to be minimized. SVR hyper-parameters were left at default values. To enable both the optimization search and make use of the full sample size available for training a generalisable regression model, the dataset was divided into a training set and a held-out test set. Bayesian optimization was first carried out using 1803 of the 2003 total participants to determine the optimal voxel size and smoothing kernel size values (allowing 22 total optimization iterations). A regression model was then trained on these 1803 images pre-processed using the identified optimal values, and tested on the 200 held-out participants in the test set (pre-processed using the same optimized parameters) to give an unbiased out-of-sample measure of age prediction performance.

A final regression model was trained on all 2003 participants (the *full model*). This allowed us to evaluate how well the optimized pre-processing parameters generalized to the independent CamCAN dataset. We compared three possible approaches for this independent validation step: (1) application of the BAHC-derived full model to age prediction on CamCAN data, (2) application of the entire Bayesian optimization framework to the CamCAN dataset followed by regression training, (3) using the BAHC-derived pre-processing parameters to process the CamCAN dataset, but training a new regression model for age prediction. In case #1 the optimized voxel size and smoothing kernel size from the BAHC dataset were applied to the CamCAN dataset. In case #2, the pre-processing parameters were optimized afresh, using only the CamCAN dataset. Case #3 is something of an intermediate iteration, generalizing the optimized pre-processing parameters, but not the trained regression model.

### Performance Stability

Finally, we performed several experiments to assess reproducibility and variability of different solutions to the classification task (i.e., young vs. old). This was done to allow inference regarding which image pre-processing parameters had the greatest impact on prediction accuracy, and to establish robustness of the model. We tested the consistency of model solutions across repetitions and participant sets, using different random seeds to create shuffled groups and burn-in points. We also varied the acquisition function of the Bayesian approach. This included comparing the results using six different acquisition functions: Expected Improvement (EI), EI per second, EI+, EI per second +, Lower Confidence Bound, and Probability of Improvement. Finally, model solutions were compared across differing values for the exploration-exploitation ratio, ranging from 10 to 90% exploration. These tests were conducted in context of voxel size and smoothing kernel size, using ranges of 1–15 mm^3^ and 1–10 mm FWHM, respectively. The acquisition function “Expected Improvement +” has a property which allows for escape from a local optimum, and returns to exploratory behavior when a region is thought to be over-exploited. The plus addition to the EI function enables this escape and refers to the additional requirement that the standard deviation of the posterior function must be less than the standard deviation of included noise multiplied by the exploration ratio.

## Results

### Classification Analyses

**FIGURE 2:**
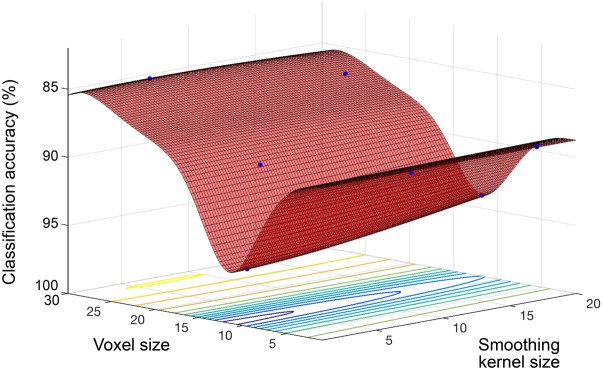
Objective function model for classification of young and old. Objective function model showing the space of voxel size and smoothing kernel values in terms of old versus young classification performance with a linear kernel SVM. The red surface is the function model and represents performance at each location. Points where the parameter space was sampled are shown (blue).

Model performance accuracy from ten-fold cross-validation was 88.1% for correct classification of neuroimaging data as either young or old (see **Table 2**). The optimized voxel size was 11.5 mm^3^ and smoothing kernel size was 2.3 mm. The performance using un-optimized values (1.5 mm^3^ voxels and 4 mm smoothing kernel) was 80.3%. The optimized model performance was significantly better than the un-optimized case (McNemar test, χ^2^= 18.76, *p* < 0.001). The parameter space exploring the expanded range of voxel size and smoothing kernel size values yielded by the model is shown in **Figure 2**.

**Table 2:** Classification performance for distinguishing young and old brains.

Un-optimized	Actual old	Actual young
Predicted old	380	120
Predicted young	77	423
**Optimized**		
Predicted old	441	59
Predicted young	60	440

### Classification Model Stability

**FIGURE 3:**
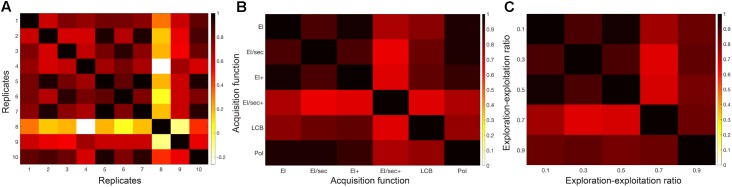
Classification model stability over varying Bayesian optimization parameters. Correlation heat maps illustrating the relationships between model objective function solutions across **(A)** replicates of the protocol, **(B)** six different acquisition functions, and **(C)** a range of exploration-exploitation ratios between 0.1 and 0.9 (where exploitation is 1-exploration).

Stability and reproducibility of model solutions were explored in the classification problem. Correlation of models in different scenarios are shown in **Figure 3**. These were; (a) across 10 repetitions of the final classification protocol, (b) produced by the use of each of six different acquisition functions, and (c) using 5 different exploration-exploitation ratios ranging between 90% exploration and 90% exploitation. In all three cases, model solutions showed high cross-correlation across replications, as well as across varying settings of the optimization process. The choice of acquisition function for Bayesian sampling and the choice of exploration-exploitation ratio of this function had little impact on final model performance. Similar models were reproducible across repetitions and in randomly shuffled participant sub-sets. This behavior implied that a stable model exists in the outlined parameter space. These observations supported our use of the default acquisition function options for the classification analysis: Expected Improvement Plus (EI+), with an exploration ratio of 0.5. We thus adopted these parameters for the regression task.

### Age Prediction Regression

**FIGURE 4:**
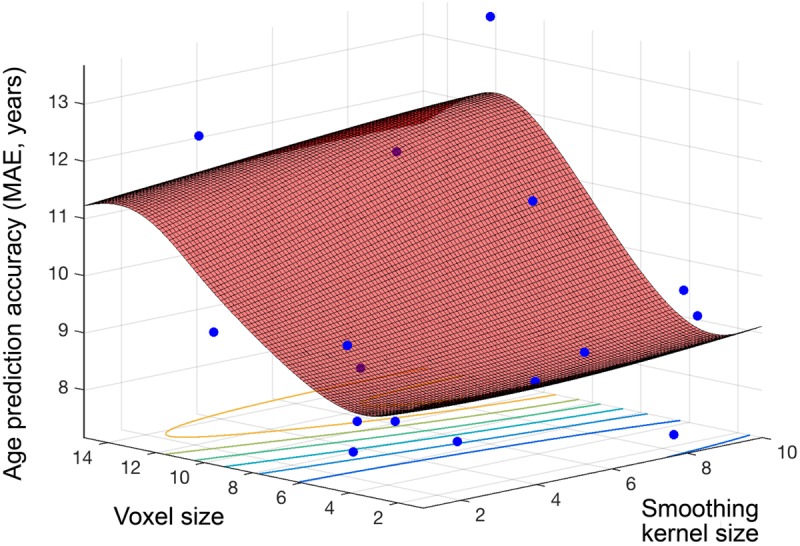
Objective function model for age prediction. Objective function model resulting from optimizing during regression for exact age prediction. The red surface is the function model and represents performance at each location. Points where the parameter space was sampled are shown (blue).

**Figure 4** shows the objective function model created during regression, when varying smoothing kernel and voxel size (1–10, 1–15), and minimizing the MAE in SVR age prediction. The lowest MAE observed for an individual sample (*N* = 80) was 7.17 years and the objective model function estimated a minimum error of 8.52 years (lowest point on the surface fitted in **Figure 4**). The estimated optimal voxel size and smoothing kernel size values were 3.73 mm^3^ and 3.68 mm respectively. Following optimization these values were used to pre-process the full dataset and train a regression model for subsequent application to other datasets.

**FIGURE 5:**
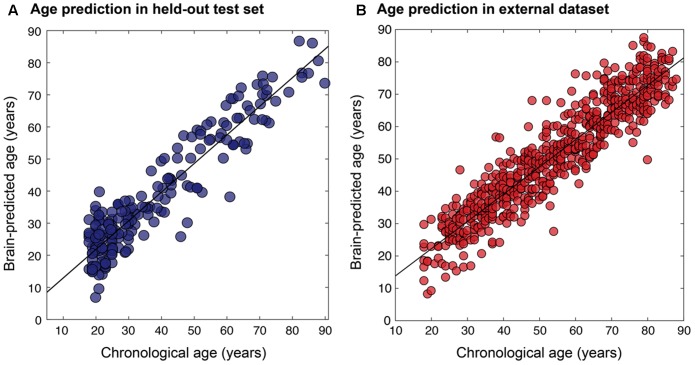
Relationship between chronological age and brain-predicted age. Chronological age (*x*-axis) plotted against brain-predicted age (*y*-axis) when testing the BAHC-trained model on **(A)** the hold-out *N* = 200 test set from BAHC (MAE = 5.08 years, *r* = 0.941, *R*^2^ = 0.89), and **(B)** on participants from the CamCAN dataset, using the pre-processing values optimized on the BAHC dataset (MAE = 6.08 years, *r* = 0.929, *R*^2^ = 0.86).

The final resulting model was applied to predict ages for the remaining 200 holdouts and achieved a MAE of 5.08 years (**Figure 5A**). This was compared to MAE = 5.48 years when using the un-optimized pre-processing values, though this value was not significantly different from the optimized case (Steiger test *p* > 0.1). The absolute error observed ranged from 0 to 22.78 years. In the hold-out set the Pearson’s correlation between predicted and true age was *r* = 0.941, with *R*^2^ = 0.89 using optimized pre-processing. Using un-optimized pre-processing parameters, we found *r* = 0.927, *R*^2^ = 0.86.

The relevance of this approach for an independent dataset (i.e., CamCAN) was considered in three different ways. (1) The BAHC-trained model was applied to the CamCAN data pre-processed with the BAHC-informed optimum voxel size and smoothing kernel size values. Here the CamCAN participants were included in the test set only. This achieved a MAE of 6.08 years, *r* = 0.929, with *R*^2^ = 0.86 (**Figure 5B**). This was an improvement compared to the performance when using un-optimized pre-processing values (voxel size = 1.5 mm^3^, smoothing kernel size = 4 mm) which resulted in MAE = 6.76 years. (2) The CamCAN data was analyzed entirely independently; the full Bayesian optimization framework was instead applied to the CamCAN data to discover new, CamCAN-specific pre-processing optima, and a new regression model was trained with 588 participants and tested on 60 participants (giving a similar training-testing ratio as used in the BAHC dataset). This resulted in optimal values of 8.41 mm^3^ for voxel size and 3.54 mm for smoothing kernel size and yielded MAE = 5.46 years, *r* = 0.91, *R*^2^ = 0.83. (3) The CamCAN cohort was pre-processed using the BAHC-informed optimum values but a new regression model was trained and tested within the CamCAN participants. This model resulted in MAE = 6.21 years, *r* = 0.89, *R*^2^ = 0.79. The model from case #2 was significantly more accurate than the model from case #3 (Steiger test, *t* = 1.67, *p* = 0.05).

## Discussion

Using Bayesian optimization, we present a conceptual improvement to conventional pipelines for distinguishing young and old brains or predicting age using neuroimaging data. The Bayesian optimization-derived optima for voxel size and smoothing kernel size showed improved performance over ‘un-optimized’ defaults for the classification of young and old brains, though performance in brain-age prediction was similar to un-optimized values. Potentially, the values previously used are relatively near the optimum thanks to the testing and experience of the researchers involved. In fact, the derived optimal smoothing kernel was very near the un-optimized value (3.68 mm vs. 4 mm). It is also important to note optima derived from the Bayesian optimization process should not be regarded as definitive, as a completely exhaustive search of the parameter space is not conducted (nor desirable due to time constraints). Our results are important as they suggest that the same pre-processing parameters are not optimal for different prediction tasks (i.e., classification vs. regression) or for different datasets (BAHC vs. CamCAN). Often, researchers will apply parameters used in one context to another. This may not necessarily be best practice, and our work shows proof-of-principle that Bayesian optimization can be used to improve image pre-processing in a principled and unbiased fashion.

Beyond optimizing model performance, our Bayesian optimization approach also allows for relative comparison of the influence of different parameters. This potentially provides novel information regarding the prediction problem at hand. For example, here we found that varying voxel size had a much greater impact on overall performance than did smoothing kernel size. This was seen in all experiments; the change in performance across the full range of values was much smaller for smoothing kernel size than voxel size, and is clearly seen in the surface plots (**Figures 1**, **2**). This suggests that in future neuroimaging pre-processing pipeline design, there is more to be gained from optimizing voxel size, rather than smoothing kernel size. The target voxel size for normalization is often not considered, though has an important impact on the degree of partial volume effects, number of simultaneous statistical tests undertaken, spatial resolution and subsequent inferences made about anatomical specificity. Our findings suggest that more weight needs to be placed on this important parameter when relating volumetric MRI data to age.

Importantly, the conclusions regarding specific optimal values are related to the particular application in which they are tested. Within this study, we observed a notable difference in the optimal voxel size for classification (11.5 mm^3^) compared to regression (3.73 mm^3^). Potentially, the more gross distinction between young and old brains benefits from a coarser resolution which increases signal-to-noise ratio, while the subtler patterns underlying gradual age-associated changes in brain structure requires finer-grained representation. Alternatively, the much larger voxel size identified here could result in better classification by reducing data dimensionality, with this size representing the optimal trade-off between representing the information and simplifying a high-dimensional feature space for more effective classification. Either way, the discrepancy in optimal voxel size between classification and regression reinforces the point that systematic evaluation of parameter specifications should be conducted case-by-case. Commonly, ‘one-size-fits-all’ is the prevailing heuristic for setting pre-processing parameters in neuroimaging analysis, where the defaults are assumed to be adequate. Our findings show that this is not necessarily the case, supporting the use of optimization techniques to improve experimental precision.

The age-prediction accuracy achieved in the BAHC dataset (MAE = 5.08 years) is comparable to the current performance seen in similar research (Konukoglu et al., 2013; Mwangi et al., 2013; Irimia et al., 2014; Cole et al., 2015, 2017b,c; Steffener et al., 2016). In these studies, pre-processing parameters are set somewhat arbitrarily (c.f. Franke et al., 2010). Here, the Bayesian optimization method offered a more principled approach. The prediction tools used here were common methods selected for computational efficiency (i.e., SVMs), in contrast with some of the studies capitalizing on state-of-the-art techniques and advanced modeling such as deep learning or Gaussian process regression (Cole et al., 2017b,c). One limitation of Bayesian optimization is the computational time needed to derive optima, which may slow its adoption with newer, computationally intensive algorithms. The duration of the optimization also depends on the process to be optimized. Here, we selected image resampling parameters as these can be computed rapidly. One important target for optimization should be image registration, however, the speed of most non-linear registration algorithms currently makes this a time-consuming goal.

We explored the generalisability of the model and the general framework. The resulting MAE in the CamCAN dataset of 6.08 years (BAHC-defined model and parameters), 5.18 years (CamCAN defined model and parameters) and 6.38 years (BAHC-defined parameters, CamCAN model) provides some interesting insights. Though the BAHC-derived model produced reasonable performance, the highest accuracy was achieved when re-optimizing and re-training within the independent cohort, using the full Bayesian optimization framework. Interestingly, the optimal smoothing kernel size was similar between the two datasets (3.68 mm vs. 3.54 mm), while there was a marked difference in optimal voxel size (3.73 mm^3^ vs. 8.41 mm^3^). Speculatively, this could be due to the specific acquisition parameters at this site, or as a result of latent sample characteristics, as truly random sampling is hard to achieve. This suggests that there is unlikely to be ground-truth optimum for a given parameter, highlighting the importance of defining such optima in a given context. This relates to another potential limitation of the approach; sufficiently large datasets are necessary for the optimization to work effectively. While this is increasingly possible due to the drive to share data and the availability of large, publicly accessible cohorts (e.g., Alzheimer’s Disease Neuroimaging Initiative, Human Connectome Project, UK Biobank), this may not be possible in certain clinical contexts, particularly regarding rarer diseases. Nevertheless, the ‘generalized’ performance of the model was still reasonable in this case, which suggests that it is incumbent on researchers to decide what constitutes sufficient prediction accuracy in each context.

Bayesian optimization is a robust and elegant way to tune pre-processing pipelines in an efficient and automated manner. In addition to the parameter optima, an additional strict, unbiased estimate for performance and generalisability is generated. The objective function model created provides detailed information on model performance and new insights can be gained from mapping the entire parameter space by enabling visualization of relationships between key components of the analysis and performance. This could allow for informed decision-making in experimental design, such as allowing for cost-benefit analysis in the case where optimal parameters only lead to marginal improvement in performance over other values which are easier, quicker, or less costly to enact. This could be critical in applications where the varied inputs represent expensive or invasive procedures, such as MRI scanning or obtaining CSF samples from lumbar punctures.

Though our analysis focused on neuroimaging pre-processing to illustrate the strengths of Bayesian optimization methods, the potential applications are far-reaching. Optimization could be applied anywhere in the experimental pipeline: in questions of experimental design, stimuli choice, data acquisition, statistical method or algorithm selection, prediction methods or final model selection. The current literature on Bayesian optimization topics applies mainly to tuning of machine learning algorithms (Snoek et al., 2012) and though machine learning is widely used in neuroscience, few studies have capitalized on this strategy to improve neuroimaging analysis or neuroimaging-based prediction (Lorenz et al., 2016, 2017). In machine learning contexts, and especially in applied multi-disciplinary fields like neuroscience where researchers may not necessarily have expertise regarding every relevant experimental parameter, more widespread use of *a priori* unbiased parameter optimization could be highly beneficial.

Our study shows the potential of Bayesian optimization to improve neuroimaging pre-processing by reducing prior assumptions, in the context of classification and regression in the context of brain aging. Future research into brain aging and other neuroscientific areas could benefit from applying principled optimization approaches to improve study sensitivity and reduce bias.

## Ethics Statement

All study data were obtained from repositories of publicly available data. These data were originally acquired with written informed consent of all study participants.

## Author Contributions

RLe and JC conceived and designed the study. RLo, RLe, and JC developed the methods. JL, RLe, and JC analyzed and interpreted the data. JL, RLo, RLe, and JC drafted and revised the manuscript.

## Conflict of Interest Statement

The authors declare that the research was conducted in the absence of any commercial or financial relationships that could be construed as a potential conflict of interest. The handling editor is currently co-organizing a Research Topic with one of the authors JC, and confirms the absence of any other collaboration.

## Footnotes

1 http://www.mrc-cbu.cam.ac.uk/datasets/camcan/

2 https://uk.mathworks.com/help/stats/bayesian-optimization-algorithm.html
